# 射频消融治疗329例肺部恶性肿瘤临床安全及疗效的研究

**DOI:** 10.3779/j.issn.1009-3419.2011.11.06

**Published:** 2011-11-20

**Authors:** 强 卢, 小飞 李, 勇 韩, 志培 张, 小龙 闫, 立军 黄

**Affiliations:** 710038 西安，第四军医大学唐都医院胸腔外科 Department of Thoracic Surgery, Tangdu Hospital, Affliated to the Fourth Military Medical University, Xi'an 710038, China

**Keywords:** 经皮射频消融, 肺肿瘤, 转移, Percutaneous radiofrequency thermal ablation, Lung neoplasms, Metastasis

## Abstract

**背景与目的:**

射频消融（radiofrequency ablation, RFA）是近年来用于无法手术的肺部恶性肿瘤及肺转移瘤治疗的替代方案。本研究旨在评估肺射频消融术的安全性和临床疗效。

**方法:**

本研究回顾性分析1999年10月-2006年7月在第四军医大学唐都医院胸腔外科进行肺部恶性肿瘤射频消融术的患者329例（其中肺部原发肿瘤237例，转移瘤92例），对其进行射频治疗后的并发症、局部进展以及1年、2年和5年总生存期的临床资料进行了研究及评价分析。

**结果:**

行射频手术的患者术后出现的并发症包括：气胸63例（19.1%），咯血14例（死亡1例，4.2%），血胸10例（3.0%），肺炎15例（4.5%）和心包填塞3例（死亡1例，0.9%），术后30天内的死亡率为0.6%，针道肿瘤种植的患者6例（1.8%）。中位无进展时间为21.6个月。1年、2年和5年总生存率分别为68.2%、35.3%和20.1%。共有78例（23.7%）患者出现后期肿瘤局部进展。肺部肿瘤原位局部进展的患者的肿瘤包块直径大多 > 4 cm; 在肿瘤局部进展方面，肿瘤 < 3 cm的患者与直径介于3 cm-4 cm的肿瘤患者相比没有明显差异，这两组患者与直径 > 4 cm的肿瘤患者间存在明显差异。

**结论:**

对于肺部恶性肿瘤来说，射频治疗是一种耐受性良好、疗效可靠安全的治疗方法。

## Abstract (en)

**Background and objective:**

In recent years, radiofrequency ablation (RFA) has been increasingly utilized as a non-surgical treatment option for patients with primary and metastatic lung tumors. The aim of this study is to evaluate the safety and therapeutic effects of RFA among patients with pulmonary neoplasms.

**Methods:**

Patients with lung tumors treated with RFA from October 1999 to July 2006 in this hospital were enrolled into the study. A total of 329 cases, 237 primary lung tumors and 92 metastatic tumors, were retrospectively analyzed and evaluated for complications, local progression, and overall survival after one, two, and five years after RFA.

**Results:**

The complications of RFA with surgery included 63 (19.1%) patients with pneumothorax, 14 (4.2%) with hemoptysis (one death), 10 (3.0%) with hemothorax, 15 (4.5%) with pneumonia, and three (0.9%) with pericardial tamponade (one death). The mortality within 30 days was 0.6%. Needle tract tumors were observed in 6 patients (1.8%). The median time for progression was 21.6 months. The overall survival rates at one, two, and five years after RFA were 68.2%, 35.3% and 20.1%, respectively. Up to 78 (23.7%) patients with tumors that exceed 4 cm developed local progression, and a significant difference was found. No significant difference was found among patients with tumors less than 3 cm and among those with 3 cm-4 cm tumors.

**Conclusion:**

RFA is a well-tolerated, reliable, and safe method for the treatment of lung malignancies.

肺癌主要分为小细胞肺癌（small cell lung cancer, SCLC）和非小细胞肺癌（non-small cell lung cancer, NSCLC）两类，由于不同的生物学特性，其治疗手段多不相同。原发性肺癌的标准治疗方法是手术治疗^[1]^，但是由于种种原因，大约80%的原发性肺癌以及其它脏器实体肿瘤肺转移的患者，无法通过手术切除治疗。在过去的几年中，射频消融（radiofrequency ablation, RFA）已渐渐成为这些非手术肺肿瘤患者的首选替代治疗方案。CT影像引导肺内肿瘤射频消融的主要优点为住院时间短、患者损伤小。研究^[2-4]^表明，射频消融具有死亡率和并发症极低的优点，但也有致命性并发症的报道^[5]^。因此射频消融的临床安全性和治疗效果仍有待商榷。本研究旨在评估肺肿瘤射频消融并发症的发生率，并对射频消融的临床治疗效果进行评价。

## 1 材料与方法

### 1.1 临床资料

回顾性分析1999年10月-2006年7月在第四军医大学唐都医院胸腔外科进行射频治疗的患者329例，其中，男性208例，女性121例，平均年龄62.1岁（20岁-80岁）。所有患者均签署了书面知情同意书，并全部进行了术后长期随访。共计有237例NSCLC患者以及92例其它肿瘤转移患者。CT共检测出483个肺内结节，其中436个结节经CT引导下行射频消融治疗，平均每例患者为1.85个结节。肿瘤结节直径 < 3 cm的患者共计253例，肿瘤结节直径介于3 cm-4 cm的患者共计102例，剩余的81例患者肿瘤结节直径 > 4 cm。肿瘤结节直径平均为（23.1±1.7）mm（9 mm-58 mm）。患者一般情况见表 1和表 2。

**1:** 非小细胞肺癌患者的一般情况（*n*=237） Characteristics of patients with non-small cell lung cancer (*n*=237)

Variables	*n*
Age (mean, yr)	67.3±11.4
Gender	
Male	156 (66%)
Female	81 (34%)
Stage	
Ⅰ	33 (14%)
Ⅱ	50 (21%)
Ⅲ	109 (46%)
Ⅳ	45 (19%)
Previous therapy	
Chemotherapy	73
Radiotherapy	52
Lung resection	112
Main reasons for no surgery	
Poor lung function	106 (45%)
Sever cardiac risk	58 (24%)
Poor performance status	40 (17%)
Refusal of surgery	33 (14%)
Length of hospital stay (month)	
Median (range)	5.2 (3-32)

**2:** 肺转移瘤患者一般情况（*n*=92） Characteristics of patients with pulmonary metastasis tumor (*n*= 92)

Variable	*n*
Age (mean, yr)	60.6±13.2
Gender	
Male	52 (5%)
Female	40 (43%)
Primary tumor	
Breast cancer	16 (17%)
Prostate cancer	18 (20%)
Liver cancer	37 (40%)
Gastrointestinal cancer	21 (23%)
Previous therapy	
Chemotherapy	28
Radiotherapy	23
Primary tumor resection	41
Main reasons for no surgery	
Poor lung function	56 (61%)
Sever cardiac risk	26 (28%)
Poor performance status	8 (10%)
Refusal of surgery	10 (11%)
Length of hospital stay (month)	
Median (range)	4.2 (3-38)

肺癌射频消融患者入选标准：①年龄介于18岁和80岁之间; ②相对早期阶段的非小细胞肺癌患者以及肺功能较差的患者（FEV1 < 1 L, FEV1% < 50%, MVV < 50%）和/或由于心脏的原因无法手术的患者; ③拒绝接受手术的患者。

### 1.2 治疗方法

治疗方案经医院伦理委员会同意，并经患者本人和家属签署知情同意书。

#### 1.2.1 射频治疗仪

射频治疗仪使用绵阳立德电子技术有限公司生产的LDRF-120S多极射频消融仪，工作频率为400 KHz。电极针为14 G套针，内套针顶端有9根分布均匀的多极细针在肿瘤内呈伞状展开，可形成3.0 cm-5.0 cm的类球形凝固区。消融范围包括肿瘤及周边0.5 cm-1.0 cm正常肺组织。

#### 1.2.2 经皮肺射频消融术操作

先行CT扫描，明确肺内包块部位; 采用局部麻醉镇静，在距肺内包块最近处胸壁切一0.5 cm的小切口，CT扫描引导下置入射频消融天线（含7个或9个可扩展针尖，连接到50 W或90 W的射频发生器上）。直径 < 3 cm的包块，进针点插入结节的中心部分，行经皮肺射频消融术。直径 > 3 cm的包块往往形状不规则，进针点插入结节最深的部分治疗后，继续调整针尖位置进行重复治疗，直至将肿瘤全部消融。治疗结束后，将锚状电极收回，拔除射频消融天线，再行CT扫描，明确包块及其周围1 cm大小范围的消融区域图像改变情况。

#### 1.2.3 并发症评估

射频治疗术后30天内均为围手术期，期间出现的相关症状均考虑为治疗相关并发症。轻微并发症标准：无后遗症，或需要轻微治疗或短时间住院观察的并发症; 主要并发症标准：需要重新入院进行治疗、护理或延长住院时间的长久的或不利的并发症，甚至是死亡。患者在术后1个月至2年期间每3个月进行1次CT复查。中位随访时间为24个月。根据实体瘤的疗效评价标准（Response Evaluation Criteria in Solid Tumors, RECIST）量化局部进展。

#### 1.2.4 疗效评价

肿瘤治疗效果参照实体肿瘤疗效评价标准，采用CT或MRI测量病变直径的变化等相关指标，客观评价肿瘤疗效。按照RECIST标准，疗效分为：完全缓解（complete response, CR）、部分缓解（partial response, PR）、疾病稳定（stable disease, SD）及疾病进展（progressive disease, PD）^[6]^。以上指标通过术后1个月至2年期间每3个月行CT复查判定，所有患者随访5年。

### 1.3 统计学处理

采用SPSS 13.0统计软件包对数据进行分析处理，两组间分类资料比较采用χ^2^检验，以*P* < 0.05为有统计学差异。

## 2 结果

### 2.1 射频消融的并发症

所有患者平均住院时间为4.5天（3天-38天）。329例患者中113例患者出现并发症（34.3%），包括气胸63例（19.1%），咯血14例（4.2%）（死亡1例），血胸10例（3.0%），肺炎15例（4.5%），心包填塞3例（0.9%）（死亡1例）; 30天的死亡率为0.6%。

射频消融4个月-6个月后出现针道种植患者6例（1.8%）; 射频消融1 h-2 h后胸痛97例，持续2 h-4 h，未进行特殊治疗。128例患者射频消融后出现发热，但温度均低于38.5 ℃，予以物理治疗或退热药物治疗后均好转。

### 2.2 射频消融患者术后疗效及生存情况

患者治疗效果明显，部分患者肿瘤坏死后经过支气管咯出，在CT上表现为空洞样改变（图 1A，图 1B）。中位无进展时间为21.6个月，1年、2年、5年总生存率分别为68.2%、35.3%和20.1%。NSCLC的1年、2年和5年生存率分别为80.1%、45.8%和24.3%;肺转移肿瘤患者的1年、2年和5年生存率分别为50.6%、30.1%和17.3%。78例（23.7%）患者出现肿瘤原位局部进展。射频消融治疗的436个结节中，253个结节直径 < 3 cm，102个结节直径为3 cm-4 cm，其余的直径均 > 4 cm。肺部肿瘤原位局部进展的患者，其肿瘤包块直径大多 > 4 cm; 在肿瘤局部进展方面，肿瘤直径 < 3 cm的患者与直径介于3 cm-4 cm的患者无明显差异（*P*=0.539），这两组患者与直径 > 4 cm的肿瘤患者之间存在明显差异（*P* < 0.05）（表 3）。

**1:**
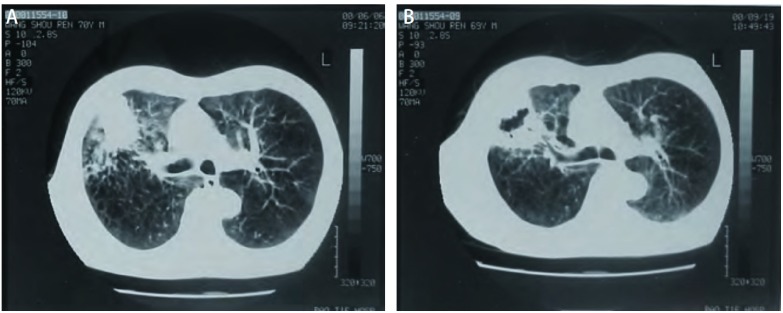
70岁老年男性患有右肺腺癌。A：术前CT影像显示可见右肺上叶4.2 cm大小肿瘤; B：术后3月可见右肺上叶空洞样改变。 70-year man with primary lung adenocarcinoma. A: CT image shows a lung tumor measuring 4.2 cm in the right upper lung before RFA. B: CT image shows a cavity in the right upper lung at 3 months after RFA. RFA: radiofrequency ablation.

**3:** 肿瘤局部进展与肿瘤直径之间的关系 The relation between sizes of tumor and local progression

Tumor diameter	*n*	No local progression (CR, PR, SD) (*n*)	Local progression (PD) (*n*)
< 3 cm	253	209 (82.61%)	44 (17.39%)
3 cm-4 cm	102	87 (85.29%)	15 (14.71%)
> 4 cm	81	58 (71.60%)	23 (28.40%)
CR: complete response; PR: partial response; SD: stable disease; PD: progressive disease. < 3 cm *vs* 3 cm-4 cm: *X*^2^=0.378, *P*=0.539; < 3 cm *vs* > 4 cm: *X*^2^=4.633, *P*=0.031; 3 cm-4 cm *vs* > 4 cm: *X*^2^=5.142, *P*=0.023.			

## 3 讨论

早在1990年即已有文献建议使用射频消融术取代无水酒精注射治疗肝肿瘤。目前射频治疗在世界范围内被广泛接受，是最为成熟的热损伤治疗技术^[7, 8]^。研究^[9]^表明，射频消融的癌肿及周边组织有明确的组织凝固性坏死，并且其病理改变分为3个阶段：炎症反应期、纤维增生期及结构恢复期。随着射频消融的广泛临床应用，其安全性和疗效已经越来越受关注。Steinke等^[10]^和Yamagami等^[11]^研究结果显示30天内的死亡率为0.4%-2.6%，而我们的研究提示30天内的死亡率为0.6%（2/329）。这些结果均表明：射频消融是一种非常安全的实践操作，但同时也会引起致命性的并发症。因此，射频消融治疗应该选择合适标准的患者，并且需要在有正规培训及有相当经验的临床医师的指导下进行操作。本研究中，1例患者因咯血死亡，CT显示其肿瘤存在明显的空洞（直径=0.8 cm），由于射频消融扩大了病灶空洞，最终导致不可抗拒的大出血而引起患者死亡。这提示我们当患者病灶部位出现空洞改变时应该谨慎。另1例患者，肿瘤位置靠近肺门，CT扫描时发现射频针已进入心包，最终患者死于心包填塞。本研究中所有出现咯血的病例几乎均是肿瘤位于叶支气管附近的中心性肺癌患者。因此，我们认为，射频消融的排除标准应包括紧邻肺门的肺血管或支气管的中央型肺部病变。

射频消融治疗后最常见的并发症是气胸，据报道^[12, 13]^其发病率约为40%-50%;在本研究中，气胸发生率约为19.1%。气胸多发生于老年患者，经胸腔闭式引流处理可很快痊愈，胸腔引流管放置的中位时间不少于24 h（范围1天-16天），长时间漏气罕见。射频消融的其它常见的并发症包括：出血、咯血和心包填塞等。研究发现，射频治疗时间超过3 h时，患者出现并发症的几率大大增加，因此，射频消融治疗时间应尽可能缩短，特别是在患者身体状况欠佳时。其它并发症包括胸部疼痛、术后发热等。胸部疼痛可能是热导致损伤胸膜神经引起的，而术后发热则可能是吸收坏死的肿瘤产物引起的。我们认为射频消融在恶性肺肿瘤的治疗中具有良好的安全性和耐受性。

本研究中患者的中位无进展时间、1年、2年、5年生存率以及肿瘤局部进展情况提示，特别是对于NSCLC患者，射频消融是明确有效的，术前术后CT对照治疗效果十分明显。肺转移瘤患者的射频消融临床疗效差，可能是由于此时的患者癌症分期较晚，所以愈后相对较差。即使是射频消融肺部恶性肿瘤成功的患者也存在肿瘤局部进展的问题，这是一个重要的需要解决的问题。文献^[14]^报道射频消融后肺癌的局部进展率从3%至38.1%不等。本研究对329例患者进行分析发现，局部进展的患者共计78例（23.7%），这表明射频消融治疗后肺内肿瘤仍可能出现局部进展，这可能是肿瘤射频治疗不完整引起的，其中肿瘤进展的部位主要位于射频消融的肺局部肺叶。除了技术需要进一步完善以外，另外可能与肿瘤大小有关，肿瘤越大复发率越高。本研究中436例射频消融的肿块，其中34例病变治疗前直径 > 4 cm，术后出现局部进展。比较其它大小的肿块，直径 > 4 cm的患者局部进展的比例相对较高，似乎较大的肿瘤应该烧蚀时间更长。Wolf等^[15]^也发现，肿瘤大小可以预测射频消融治疗后复发的可能性; 肿瘤直径≤3 cm，完全消融后患者预后最好; 肿瘤直径 > 3 cm，消融术后有复发的倾向。而本研究提示，在肿瘤局部进展方面，肿瘤 < 3 cm的患者与直径介于3 cm-4 cm的患者没有统计学差异（*P*=0.539）。我们认为肿瘤直径介于3 cm-4 cm的肿块可以考虑作为射频治疗的标准之一。肺癌的5年生存率极低，不到15%^[16, 17]^。本研究提示，射频消融可提高无法手术的NSCLC及转移性肺癌的患者生存率并降低死亡率。由于可以对局部进展的肿瘤进行再次消融治疗，无法手术的NSCLC患者的5年生存率可达到24.3%，肺转移肿瘤患者的5年生存率可达到17.3%，临床治疗效果明显。

肺恶性肿瘤患者在诊断及治疗过程中常常出现肿瘤的播种转移。Akeboshi等^[18]^发现在9, 738例肺穿刺活检中有6例肿瘤播种现象的发生，恶性肿瘤患者的种植危险度为0.061%左右。本研究跟踪观察发现射频消融后肿瘤种植发病率约为1.8%。因此，与经皮肺活检相比较，射频消融胸部创伤稍大，射频消融引起的恶性肿瘤播种的风险更高，且目前临床使用的射频消融仪无针道消融功能，无法避免针道转移。这种肿瘤的播种风险是随机且可以接受的，并可能随射频消融次数的减少而减少。

该大样本的研究提示射频消融在治疗肺内恶性肿瘤中的效果令人满意，且其安全性和耐受性都较好。为了避免潜在的致命并发症，操作足够熟练的工作人员以及细致的患者选择都对射频治疗肺内肿瘤的临床工作有巨大帮助。
